# A Current Overview of Quality-of-Life Research in Chronic Myeloid Leukemia (CML)

**DOI:** 10.3390/cancers18071133

**Published:** 2026-04-01

**Authors:** Stefan Deneberg

**Affiliations:** Karolinska Institutet, Institutionen för Medicin Huddinge, ME Hematologi, Karolinska University Hospital, 141 57 Huddinge, Sweden; stefan.deneberg@regionstockholm.se

**Keywords:** chronic myeloid leukemia, health-related quality of life, patient-reported outcomes, fatigue, tyrosine kinase inhibitors, treatment-free remission, dose reduction, health utilities

## Abstract

Background/Objectives: The introduction of tyrosine kinase inhibitors has transformed chronic myeloid leukemia into a chronic condition with near-normal life expectancy, shifting clinical priorities toward long-term tolerability, symptom burden, and health-related quality of life. This review aims to synthesize current evidence on patient-reported outcomes and quality of life in chronic myeloid leukemia, with particular focus on fatigue, differences across therapies, associations with molecular response and treatment-free remission, and health utility outcomes. Methods: A narrative review of published clinical trials, observational studies, and patient-reported outcome research in chronic myeloid leukemia during the tyrosine kinase inhibitor era was conducted. Evidence from generic, cancer-specific, and disease-specific quality-of-life instruments was examined, together with studies evaluating dose reduction, treatment discontinuation, and survivorship outcomes. Results: Across studies, patients with chronic myeloid leukemia experience persistent impairments in quality of life compared with the general population despite effective disease control. Fatigue consistently emerges as the dominant symptom associated with functional limitation and reduced well-being. Comparative analyses across tyrosine kinase inhibitors demonstrate generally modest and inconsistent differences in global quality of life. Deeper molecular responses, dose reduction, and treatment-free remission are commonly associated with stabilization or improvement in patient-reported outcomes, although symptoms may persist in a subset of patients. Methodological limitations include heterogeneity of instruments, predominance of cross-sectional designs, and lack of established minimal important differences for disease-specific measures. Conclusions: In the modern treatment era, chronic myeloid leukemia is characterized by excellent survival but an ongoing quality-of-life burden, largely driven by fatigue. Future research should prioritize longitudinal patient-reported outcome collection, methodological standardization, and development of clinically meaningful thresholds to better align disease control with patient-centered outcomes.

## Simple Summary

Advances in treatment have transformed chronic myeloid leukemia into a long-term condition with near-normal life expectancy for most patients. As a result, how patients feel and function in daily life has become as important as controlling the disease itself. This review summarizes current research on quality of life in people living with chronic myeloid leukemia, with a particular focus on symptoms, treatment side effects, and patient-reported experiences. The findings show that many patients continue to experience reduced quality of life despite excellent disease control, with fatigue being the most common and impactful symptom. Differences in quality of life between available treatments are generally small, while strategies such as dose reduction or stopping treatment in selected patients may improve well-being. By highlighting consistent findings and remaining gaps in knowledge, this review aims to guide future research and support more patient-centered care in long-term cancer treatment.

## 1. Introduction

Chronic myeloid leukemia has undergone a profound therapeutic transformation since the introduction of *BCR::ABL1*-targeted TKIs [1]. Long-term survival for patients diagnosed in the chronic phase now approaches that of the general population, fundamentally changing expectations for disease management [2,3]. As a consequence, the clinical focus has broadened from preventing disease progression to include optimization of long-term tolerability, adherence, and quality of life.

Most patients with CML remain on lifelong TKI therapy, while a subset achieves sustained deep molecular responses that allow treatment discontinuation within structured TFR programs [4]. In both scenarios, patients experience prolonged exposure to therapy-related toxicities or, in the case of TFR, ongoing surveillance and uncertainty regarding relapse. Traditional clinical endpoints such as molecular response or survival do not adequately capture these patient experiences [5]. Patient-reported outcomes have therefore emerged as essential tools to complement clinical metrics and inform both research and routine care [6,7].

This review synthesizes current evidence on HRQoL and PROs in CML during the TKI era. I focus on consistent findings across studies, examine differences across therapies and disease states, and highlight key methodological limitations and unmet needs relevant to future research and clinical practice.

## 2. Methods

This narrative review summarizes the published literature on health-related quality of life (HRQoL) and patient-reported outcomes (PROs) in adult chronic myeloid leukemia (CML) in the tyrosine kinase inhibitor (TKI) era. Relevant studies were identified through searches of major biomedical databases, including PubMed/MEDLINE and reference lists of key articles. The search focused on publications addressing quality of life, patient-reported outcomes, fatigue, symptom burden, treatment comparisons between TKIs, and treatment-free remission in CML.

The search primarily covered the literature published during the TKI era, with emphasis on studies published from approximately 2000 onward. Search terms included combinations of “chronic myeloid leukemia”, “quality of life”, “patient-reported outcomes”, “fatigue”, “health utility”, and “treatment-free remission”.

Both clinical trials and observational studies reporting HRQoL or PRO data in adult patients with CML were considered relevant. Studies were selected based on their relevance to key themes of the review, including symptom burden, fatigue, comparative tolerability of different TKIs, associations between molecular response and quality of life, and outcomes related to dose reduction or treatment continuation.

Given the narrative nature of this review, a formal systematic screening process or quantitative meta-analysis was not performed. Instead, the aim was to synthesize and critically discuss representative studies that have contributed substantially to the current understanding of HRQoL and PROs in CML.

As this work represents a narrative review of previously published studies, treatment regimens, dosing strategies, and adherence assessments varied across the included reports according to the design of the original trials or observational studies. No attempt was made to standardize treatment exposure across studies; rather, the review summarizes the evidence as reported in the primary literature.

## 3. Results

### 3.1. Patient-Reported Outcome Measures (PROMs) in Chronic Myeloid Leukemia: Methodological Considerations

Patient-reported outcome measures are indispensable for capturing the lived experience of patients with chronic myeloid leukemia in the tyrosine kinase inhibitor era. However, the diversity of PROMs used in CML research reflects both the multidimensional nature of health-related quality of life and the absence of consensus regarding optimal instrument selection. PROMs used in CML can broadly be categorized into generic, cancer-specific, and disease-specific instruments, each with distinct strengths and limitations. Table 1 summarizes the key instruments used in CML HRQoL research.

#### 3.1.1. Generic Instruments

Generic instruments such as the SF-36 and EQ-5D facilitate comparisons with the general population and across disease areas. The SF-36 provides detailed profiling of physical and mental health domains, enabling identification of specific areas of impairment relative to population norms [8]. In CML, SF-36 assessments show consistent impairments in physical functioning and vitality, while mental health domains are relatively preserved, with greater variation observed in older patients and those with significant comorbidity [9,10,11,12,13]. However, generic instruments may lack sensitivity to treatment-related symptoms specific to CML, particularly low-grade chronic toxicities that accumulate over years of therapy.

The EQ-5D plays a central role in health-economic evaluations and policy decision-making due to its ability to generate utility values for quality-adjusted life year calculations [14]. In CML, EQ-5D studies have demonstrated meaningful differences between patients on long-term TKI therapy and those in treatment-free remission [15]. Nevertheless, ceiling effects are common, particularly in younger or otherwise healthy patients, potentially underestimating the burden of persistent symptoms such as fatigue or musculoskeletal discomfort. As a result, EQ-5D utility values may inadequately reflect clinically relevant differences in daily functioning and well-being.

**Table 1:** Patient-reported outcome instruments. Abbreviations: EORTC QLQ-C30—European Organisation for Research and Treatment of Cancer Quality of Life Questionnaire-Core 30; EORTC QLQ-CML24—European Organisation for Research and Treatment of Cancer Qu ality of Life Questionnaire–Chronic Myeloid Leukemia 24; EQ-5D—EuroQol 5-Dimension; EQ-5D-5L—EuroQol 5-Dimension 5-Level Version; FACT-Leu—Functional Assessment of Cancer Therapy–Leukemia; FACIT-GP5—Functional Assessment of Chronic Illness Therapy–General Physical Item 5; MDASI-CML—MD Anderson Symptom Inventory–Chronic Myeloid Leukemia; PGIC-CML—Patient Global Impression of Change–Chronic Myeloid Leukemia; SF-36—Short Form-36 Health Survey; WPAI-CML—Work Productivity and Activity Impairment Questionnaire–Chronic Myeloid Leukemia; HRQoL—health-related quality of life.

Instrument	Category	Main Domains Assessed	Major Strengths	References
EORTC QLQ-C30	Cancer-specific	Global health, functional scales, symptom burden	Broad oncologic comparability; widely used in trials	[9]
EORTC QLQ-CML24	Disease-specific	Treatment burden, daily life impact, worry, body image, satisfaction	Designed specifically for CML; robust validation	[11,12]
EQ-5D/EQ-5D-5L	Generic/health utility	Mobility, self-care, usual activities, pain/discomfort, anxiety/depression; utility index	Essential for health-economic evaluation and QALY estimation	[6,7]
FACT-Leu	Leukemia-specific	Physical, emotional, functional well-being; leukemia-related concerns	Validated in CML; captures leukemia-specific concerns	[10]
FACIT-GP5	Single-item global toxicity burden	Overall treatment side-effect burden	Efficient summary measure; increasingly used in TKI trials	[16]
MDASI-CML	Disease-specific symptom inventory	Fatigue, pain, emotional distress, cognitive symptoms, interference with daily activities	Sensitive to TKI-related symptom burden; practical for longitudinal monitoring	[14]
PGIC-CML	Global impression measure	Patient-perceived change in overall condition	Useful anchor for minimal important difference determination	[15]
SF-36	Generic HRQoL	Physical functioning, vitality, mental health, social and role functioning	Enables comparison with general population; detailed domain profiling	[1]
WPAI-CML	Work productivity instrument	Work time missed, impairment while working, activity impairment	Captures socioeconomic and functional impact; relevant for younger patients	[16]

#### 3.1.2. Cancer-Specific Instruments

Cancer-specific instruments, including the EORTC QLQ-C30 and FACT-Leu, FACIT capture broader oncologic domains such as role functioning, emotional well-being, and social participation [16,17,18,19]. These instruments have been widely used in both clinical trials and real-world CML studies and allow benchmarking against other cancer populations. However, their focus on acute cancer-related symptoms may limit sensitivity in a disease characterized by long-term oral therapy and chronic low-grade toxicity rather than intensive short-term treatment.

#### 3.1.3. Disease-Specific Instruments in CML

The development of disease-specific instruments represents an important step toward addressing these limitations. The EORTC QLQ-CML24 was developed in the early 2010s and first published in 2014 as a disease-specific module to complement the EORTC QLQ-C30 in patients with chronic myeloid leukemia, specifically designed to complement the QLQ-C30 by capturing CML- and TKI-specific issues, including treatment burden, worry, body image, and satisfaction with care [20]. Validation studies have demonstrated robust psychometric properties across international cohorts, supporting its use in both research and clinical contexts. Importantly, reference values are now available, facilitating interpretation of cross-sectional findings [21]. The MD Anderson Symptom Inventory–CML (MDASI-CML) is a disease-specific patient-reported outcome instrument developed to assess symptom burden and its impact on daily functioning in patients with chronic myeloid leukemia. It is based on the core MDASI framework and includes additional items relevant to CML and tyrosine kinase inhibitor-related toxicity, enabling more sensitive detection of disease- and treatment-specific symptoms [22].

#### 3.1.4. CML-Adapted General Instruments

The Patient Global Impression of Change (PGIC) is a single-item, patient-reported measure that captures a patient’s overall perception of change in health status over time. In CML, PGIC has been used as an anchor to interpret changes in HRQoL and symptom burden, particularly in studies evaluating treatment effects and minimal important differences [23], while the Work Productivity and Activity Impairment Questionnaire (WPAI) is a validated instrument used to assess the impact of health problems on work productivity and daily activities. In CML, adapted versions (WPAI-CML) have been used to quantify absenteeism, presenteeism, and overall activity impairment associated with disease and treatment [24].

Despite these advances, several methodological challenges remain. One limitation is the absence of established minimal clinically important differences for the QLQ-CML24. Without validated thresholds for clinically meaningful change, interpretation of longitudinal results and between-group differences remains challenging. Many studies rely on statistical significance alone, which may overestimate the clinical relevance of small score changes in large cohorts or fail to detect meaningful changes in smaller studies.

Another challenge is the predominance of cross-sectional designs in CML HRQoL research. While cross-sectional studies provide valuable snapshots of symptom burden and functioning, they do not capture individual trajectories over time, particularly during key transitions such as dose adjustments or treatment discontinuation. Longitudinal PROM collection remains underutilized, especially outside clinical trials.

Finally, PROM selection is often driven by feasibility rather than conceptual alignment with study objectives. This has resulted in substantial heterogeneity across studies, limiting synthesis and comparison. Future research would benefit from greater standardization of PROM use, potentially through the development of core outcome sets for CML that incorporate both generic and disease-specific measures.

### 3.2. HRQoL Compared with the General Population

Multiple cross-sectional and controlled studies consistently show that patients with CML experience impaired HRQoL compared with age- and sex-matched individuals from the general population [9,10,25]. Deficits are most pronounced in physical and emotional functioning, including fatigue.

Subgroup analyses reveal that younger patients and women report the greatest impairments. Younger patients often experience substantial limitations in work and social roles, while women consistently report worse HRQoL compared with men when benchmarked against population norms [9]. This pattern is similar to HRQoL findings in other cancers with generally shorter treatment duration [26,27], and underscores the importance of considering demographic and life-stage factors when interpreting HRQoL outcomes in CML. Importantly, the persistent HRQoL impairment observed in CML contrasts with the substantial improvements in survival achieved in the TKI era. While many patients attain near-normal life expectancy, long-term treatment is frequently associated with chronic low-grade symptoms that may accumulate over time and negatively affect daily functioning. Fatigue, musculoskeletal complaints, and gastrointestinal symptoms are among the most commonly reported issues and may contribute to reduced physical and social well-being.

Furthermore, comparisons with the general population may underestimate the true burden experienced by patients, as individuals with CML often adapt to chronic symptoms and adjust their expectations over time. This phenomenon, sometimes referred to as response shift, may lead to relatively stable HRQoL scores despite ongoing symptom burden [28].

### 3.3. Symptom Burden in Chronic Myeloid Leukemia: Fatigue as a Multidimensional Survivorship Outcome

Among all symptoms reported by patients with CML receiving TKI therapy, fatigue consistently emerges as the most prevalent, persistent, and impactful [11]. Fatigue in CML is not merely a subjective complaint but a multidimensional construct encompassing physical exhaustion, cognitive impairment, and reduced capacity for social and occupational functioning. Across studies using diverse PROMs, fatigue demonstrates the strongest association with overall HRQoL, often exceeding the impact of other commonly reported symptoms [10,11].

#### 3.3.1. Physical Fatigue

Physical fatigue manifests as reduced stamina, increased need for rest, and diminished capacity for sustained activity. Objective assessments using accelerometry corroborate patient-reported fatigue, showing that fatigued patients engage in significantly less daily physical activity compared with matched controls [29]. These findings underscore that fatigue in CML has tangible functional consequences that extend beyond perceived discomfort.

#### 3.3.2. Cognitive Fatigue

Cognitive fatigue, including difficulties with concentration, memory, and mental endurance, is recognized as an important component of the symptom burden. Although less frequently measured explicitly, impairments in cognitive functioning are reflected in reduced scores on cognitive domains of cancer-specific PROMs and contribute to difficulties in work performance and daily decision-making. There have been case reports and small retrospective studies published in meeting-abstract format, implicating memory impairment associated with TKI use [30,31,32,33,34], as well as depression [10], but there are also reports of improvement in cognitive functions [35,36]. Dissecting possible specific neurological side effects from other dimensions of fatigue is challenging using regular PROM instruments, and specific studies are warranted.

#### 3.3.3. Etiology of Fatigue

The etiology of fatigue in CML is multifactorial. Although better HRQoL is associated with deeper responses [37,38], fatigue often persists despite deep molecular remission, indicating that disease control alone is insufficient to resolve symptoms [9,39]. Fatigue is a recognized side effect of TKI therapy [40], but comorbid conditions, polypharmacy, physical deconditioning, and psychosocial factors may play contributory roles. Inflammatory mechanisms have been proposed as contributors to cancer-related fatigue, including in long-term survivors, although definitive biological correlates remain incompletely understood and inconsistent across studies [7,41].

The biological mechanisms underlying cancer-related fatigue are complex and likely multifactorial. Increasing evidence suggests that inflammatory pathways play a central role, with elevated levels of pro-inflammatory cytokines such as interleukin-6 and tumor necrosis factor-alpha being associated with fatigue severity [41]. In addition, immune dysregulation related to both the malignancy itself and long-term TKI therapy may contribute to persistent fatigue symptoms [42].

Neuroendocrine alterations have also been implicated, particularly dysregulation of the hypothalamic–pituitary–adrenal (HPA) axis, which may affect stress responses, energy regulation, and circadian rhythms. Furthermore, fatigue may be influenced by central nervous system mechanisms, including altered neurotransmitter signaling and changes in neural connectivity involved in motivation and cognitive processing.

Importantly, these biological factors often interact with psychological and behavioral components such as sleep disturbances, reduced physical activity, and mood disorders, contributing to the persistence of fatigue even in patients with well-controlled disease.

Despite its central importance, fatigue remains under-recognized and undertreated in routine clinical practice. Clinical encounters often prioritize laboratory and molecular results, leaving limited time for systematic symptom assessment. PROM-based fatigue measures offer an opportunity to identify patients with clinically meaningful impairment and to guide supportive care interventions, including physical activity programs, dose optimization, and psychosocial support, although evidence for such interventions is still scarce.

Taken together, existing evidence positions fatigue as a dominant survivorship issue in CML. Addressing fatigue requires a shift from viewing it as an unavoidable side effect toward recognizing it as a modifiable outcome deserving targeted intervention and systematic evaluation.

### 3.4. HRQoL Across Tyrosine Kinase Inhibitors

Comparative analyses of HRQoL across different TKIs have been conducted in both randomized trials and real-world settings. In first-line therapy, propensity-matched and randomized studies comparing imatinib with second-generation TKIs such as Dasatinib or bosutinib report small advantages for newer agents in selected disease-specific domains, including impact on daily life and symptom burden. However, these differences are often more pronounced for younger patients and not consistently observed across generic HRQoL measures [43,44].

PRO analyses from the trial demonstrated preservation or modest improvement of HRQoL with both bosutinib and imatinib over 12 months, without statistically significant between-group differences [44]. Similarly, longitudinal analyses suggest that while toxicity profiles differ across TKIs, these differences translate into relatively modest net effects on global HRQoL.

The EnestND trial comparing nilotinib to imatinib in newly diagnosed patients. HRQoL was assessed using the Functional Assessment of Cancer Therapy–Leukemia (FACT-Leu) and the Short Form 36 Health Survey (SF-36) tools. Of patients remaining on study treatment, there were no differences between study arms in either instrument, and overall scores were comparable to the general US population. However, a cohort-level analysis with imputation according to the reason of discontinuation in the FACT-Leu subscale revealed nominally lower scores in the imatinib arm, largely due to discontinuation of treatment [45]. The latter analysis is, however, only published in abstract format and not peer-reviewed, and findings should be interpreted with caution.

HRQoL data from the ASCEMBL trial comparing asciminib to bosutinib in patients with CML-CP previously treated with ≥2 TKIs showed a trend of improvement in the MDASI-CML and EQ-5D-5L scores in the asciminib arm, including fatigue, whereas there was a worsening of gastrointestinal symptoms in the bosutinib arm. The changes were deemed not clinically significant except for worsening of diarrhea in the bosutinib arm [46].

Overall, current evidence indicates that HRQoL differences between TKIs are generally small and inconsistent, limiting the utility of HRQoL as a primary differentiator for first-line TKI selection.

### 3.5. Molecular Response, Dose Reduction, and Treatment-Free Remission

There are several studies showing that HRQoL at baseline is improved during TKI therapy, both in first-line and later-line settings [47,48,49]. There is also an association between better molecular outcomes during therapy and lower fatigue levels on a group level [11]. Furthermore, HRQoL levels at diagnosis have been shown to be a prognostic factor for achieving MR even in multivariable analyses [49,50,51].

However, these findings should not be interpreted as evidence that pursuing deeper molecular responses necessarily translates into improved HRQoL. On an individual level, the association between molecular response and HRQoL is inconsistent. Symptoms such as fatigue, musculoskeletal pain, and gastrointestinal discomfort frequently persist even among patients with sustained molecular remission. This disconnect highlights an important conceptual distinction between biological disease control and symptomatic recovery. From a patient perspective, remission defined solely by molecular criteria may not equate to restoration of normal functioning or well-being. Also, much of the available evidence is based on observational or post hoc analyses only published as meeting abstracts [50,51], and the relationship between molecular response and HRQoL may be influenced by confounding factors. For example, patients achieving deeper responses often differ from others with respect to treatment duration, baseline characteristics, comorbidity burden, and overall treatment tolerance. These factors may independently affect HRQoL, making it difficult to establish a direct causal relationship between molecular response and patient-reported outcomes.

Reducing TKI dose is an appealing and often applied strategy to reduce side effects and improve tolerability, and supposedly also QoL. Several dose reduction studies have been published [52,53,54], but few of them collected HRQoL data [55]. An ongoing study capturing longitudinal PROM data in dose reduction patients is showing signs of symptom improvement in an interim analysis of 146 patients [56].

Treatment-free remission represents a particularly informative model for examining the relationship between disease control and HRQoL. Successful TFR eliminates ongoing exposure to TKI-related toxicities and has been associated with improvements in several HRQoL domains, particularly fatigue, physical functioning, and global quality of life [57,58,59]. Similarly, health utility analyses demonstrate higher utility values among patients where dose reductions and/or TFR are achievable compared with those on continued therapy, suggesting meaningful gains at both individual and population levels [60].

At the same time, TFR introduces unique psychological challenges. The transition from continuous therapy to active surveillance can be accompanied by anxiety related to molecular relapse, particularly in the early discontinuation phase. Some patients experience heightened vigilance regarding laboratory results, with transient declines in emotional well-being despite physical symptom improvement. In addition, a subset of patients developed withdrawal syndromes characterized by musculoskeletal pain and stiffness, which may temporarily offset HRQoL gains after treatment discontinuation [61,62,63].

These findings underscore that TFR is not a uniformly positive experience for all patients and that HRQoL trajectories during TFR are heterogeneous; most available data are derived from selected patient populations enrolled in clinical trials or specialized programs, limiting generalizability. Even so, most publications view achievement of dose reduction and TFR as positive regarding QoL outcomes [64].

Beyond TFR, survivorship psychology in CML is an underexplored domain. Many patients experience a prolonged identity as “chronically ill” despite an excellent prognosis, shaped by daily medication, regular monitoring, and persistent symptoms. Younger patients may face particular challenges related to career planning, family life, and long-term uncertainty. These psychosocial dimensions are incompletely captured by existing PROMs and warrant greater attention in future research.

### 3.6. Health Utilities and Quality-Adjusted Life Expectancy

Health utility studies provide a population-level perspective on the burden of CML. Despite near-normal life expectancy, quality-adjusted life expectancy remains substantially reduced, indicating that morbidity rather than mortality dominates the long-term disease burden [25]. Utility losses are closely linked to fatigue and functional impairment [15], reinforcing the importance of symptom control and supportive care in optimizing long-term outcomes.

These findings have important implications for health-economic evaluations and policy decisions, particularly in the context of lifelong therapy and TFR strategies.

### 3.7. Clinical Implications for Routine CML Care

The evidence summarized in this review has important implications for the routine clinical management of CML. First, it reinforces that molecular monitoring alone is insufficient to capture treatment impact from the patient perspective. Systematic assessment of HRQoL and symptom burden should be considered an integral component of long-term CML care, particularly for patients reporting persistent fatigue or functional impairment.

Second, the modest and inconsistent HRQoL differences observed between TKIs suggest that expectations of large quality-of-life gains should not drive first-line TKI selection in isolation. Instead, treatment decisions should incorporate individual toxicity profiles, comorbidities, patient preferences, and lifestyle considerations.

Third, whether dose optimization or switching therapy may yield meaningful HRQoL improvements even in the absence of changes in molecular response is not systematically explored, but used with judgment, it may be a viable option even if evidence is scarce. Current ELN and NCCN guidance supports dose optimization and careful adverse-event management, and recommends switching TKIs when intolerance or serious toxicity occurs; changes should not be made solely for minor or poorly characterized reasons [5,65]. Thresholds for TKI switching due to HRQoL reasons are difficult to define and, in the absence of hard evidence, are currently best decided individually after careful consideration on a case-by-case basis. Importantly, the few existing longitudinal PROM-studies show that many individual side effects of TKI therapy improve with time and depth of response [38], some HRQoL issues may persist longitudinally [44], notably, fatigue [9,66].

Fourth, TFR should be considered not only as a strategy to reduce treatment burden but also as a potential means to improve HRQoL in appropriately selected patients. Discussions regarding TFR should explicitly address potential psychological challenges, including anxiety and withdrawal symptoms. Incorporating PROMs into TFR programs may help identify patients requiring additional support during treatment discontinuation and surveillance.

Finally, I would like to highlight fatigue as a key QoL problem for CML patients. Persistent fatigue as well as other chronic low-grade toxicities may reduce motivation to maintain strict adherence to daily oral therapy, particularly when patients perceive limited symptomatic benefit from continued treatment. In this context, fatigue represents not only a quality-of-life issue but also a potential barrier to optimal disease management. To address this, non-medical interventions may be important. One small study showed that the use of cognitive behavioral therapy could improve fatigue in CML patients [67], raising hope that non-medical interventions could be effective. Structured physical activity programs, management of comorbid conditions, review of concomitant medications, and psychosocial support may all contribute to symptom improvement and are used in selected patients, albeit with limited supporting evidence. PROM-based screening can help identify patients most likely to benefit from such interventions.

It is encouraging that recent guideline recommendations [5] increasingly emphasize quality of life, long-term tolerability, and patient preferences as key goals of CML management. Translating these principles into practice will require practical frameworks for PROM integration, including the selection of feasible instruments, the interpretation of results, and the incorporation into clinical decision-making.

### 3.8. Methodological Limitations and Future Research Needs

When interpreting the available literature on health-related quality of life in chronic myeloid leukemia, several important methodological limitations should be considered.

First, a substantial proportion of HRQoL data originates from clinical trials with highly selected patient populations. These cohorts often exclude individuals with significant comorbidities, advanced age, or poor performance status, and may therefore not fully reflect the broader population of patients treated in routine clinical practice. As a result, HRQoL outcomes reported in clinical trials may overestimate the well-being of patients in real-world settings.

Second, missing patient-reported outcome data represent a well-recognized challenge in HRQoL research. Completion rates vary across studies (Table 2), and patients experiencing greater symptom burden or treatment-related toxicity may be less likely to complete questionnaires. This introduces the potential for response bias and may lead to systematic underestimation of symptom severity in longitudinal analyses [68]. In addition, differences in data collection methods and follow-up schedules further complicate comparisons across studies.

Third, many studies evaluating HRQoL in CML are cross-sectional in design. While such studies provide valuable insight into symptom burden and patient experience, they do not allow causal relationships to be established between treatment exposure, molecular response, and HRQoL outcomes. Longitudinal studies with repeated PROM assessments are therefore needed, particularly around key clinical transitions such as dose modifications, treatment switching, and treatment-free remission.

Fourth, heterogeneity in HRQoL measurement instruments represents a major barrier to the synthesis of the literature. Generic instruments, cancer-specific tools, and disease-specific questionnaires capture overlapping but distinct aspects of patient experience, limiting direct comparability across trials and real-world studies. Development of a standardized core outcome set for CML, incorporating both generic and disease-specific PROMs, would facilitate more consistent data collection and interpretation.

Fifth, the absence of established minimal important differences for many HRQoL instruments used in CML studies complicates the interpretation of both longitudinal changes and between-group differences. Without validated MID thresholds, it remains difficult to determine whether observed differences are clinically meaningful. Future research should prioritize anchor-based approaches to MID determination, ideally incorporating patient global impression of change measures.

Beyond these methodological considerations, future research should focus on translating HRQoL findings into clinically actionable strategies. Prospective studies are needed to determine how PRO data can inform treatment decisions, including selection between tyrosine kinase inhibitors, dose adjustments, and decisions regarding treatment discontinuation. In particular, the role of HRQoL in balancing treatment efficacy and tolerability should be further clarified.

In parallel, there is a clear need for interventional studies targeting symptom burden, especially fatigue, which remains the most consistently reported and impactful symptom in CML. While its prevalence is well documented, evidence on effective management strategies remains limited. Future studies should therefore evaluate both pharmacological approaches, such as dose optimization or switching between TKIs, and non-pharmacological interventions, including structured physical activity, psychosocial support, and behavioral interventions.

In addition, long-term survivorship in CML requires greater attention. As patients increasingly achieve near-normal life expectancy, research should expand beyond traditional clinical outcomes to include domains such as cognitive function, work capacity, social participation, and sexual health. These aspects are particularly relevant for younger patients and may have a substantial impact on overall well-being.

Finally, advances in digital health and real-world data offer new opportunities for HRQoL research. Digital PROM platforms and integration into electronic health records may enable more frequent and systematic assessment of patient-reported outcomes, allowing earlier identification of patients at risk of deterioration and more timely intervention. However, successful implementation will require careful consideration of feasibility, data interpretation, and clinician workload.

## 4. Conclusions

In the TKI era, chronic myeloid leukemia is characterized by excellent survival but persistent impairment in quality of life. Fatigue is the dominant driver of reduced HRQoL, while differences between TKIs appear modest and inconsistent. Deeper molecular responses, dose reduction and treatment-free remission are generally associated with improved patient-reported outcomes but do not fully eliminate symptom burden for all patients. Future research should prioritize longitudinal PROM collection, standardization of instruments, and development of clinically meaningful thresholds for CML-specific measures to better align disease control with patient-centered outcomes.

## Figures and Tables

**Table 2:** Prospective randomized trials reporting patient-reported outcomes. QoL = quality of life. PRO = patient-reported outcomes. HRQoL = health-related QoL.

Study Name	Line of Therapy/Setting	Design	Sample Size	PRO Completion Rate	QoL/PRO Instrument(s)	References	Key HRQoL Findings
BFORE	First-line bosutinib vs. imatinib	Phase 3 randomized trial	487	12 months: bosutinib arm 78.9%, imatinib arm 79.3%	FACT-Leu; EQ-5D	[44]	HRQoL preserved or modestly improved in both arms over 12 months; no significant between-group differences.
ENESTnd	First-line nilotinib vs. imatinib	Phase 3 randomized trial	846	Not reported—missing data imputed	FACT-Leu; SF-36	[45]	Comparable HRQoL among patients remaining on treatment; imputation suggested nominally worse FACT-Leu scores with imatinib due to discontinuation.
ASCEMBL	≥2 prior TKIs; asciminib vs. bosutinib	Phase 3 randomized trial	233	48 weeks: asciminib arm: 84.8% bosutinib arm: 95.5%	MDASI-CML; EQ-5D-5L; PGIC-CML; WPAI-CML	[46]	Trend toward improved fatigue and global scores with asciminib; worsening gastrointestinal symptoms with bosutinib; mostly not clinically significant.
ASC4FIRST	First-line asciminib vs. imatinib or 2nd-generation TKIs	Phase 3 randomized trial	405	96 weeks: asciminib arm: 75.5 and 76.2%; investigators-choice arm: 65.2 and 65.2% for EORTC QLQ-C30 and EORTC QLQ-CML24, respectively	EORTC QLQ-C30; EORTC QLQ-CML24 (PRO-CTCAE; FACIT-GP5 not yet reported)	[47]	Generally, larger improvements in EORTC-QLQ-C30 and QLQ-CML24 scales for asciminib over imatinib. Less but still significantly larger improvements also in asciminib compared to second-generation TKIs.
IRIS	First-line imatinib vs. interferon alpha and low-dose cytarabine	Phase 3 randomized trial	1049	12 months: imatinib arm: 82%; interferon + AraC arm 62%	FACT-BRM; EQ-5D	[48]	Decrease in physical function and well-being in the control arm, maintained level in the imatinib arm.

## Data Availability

No new data were created or analyzed in this study.

## Abbreviations

The following abbreviations are used in this manuscript:

*BCR::ABL1*	Fusion oncogene driving chronic myeloid leukemia
CML	Chronic myeloid leukemia
CML-CP	Chronic myeloid leukemia, chronic phase
EFS	Event-free survival
ELN	European LeukemiaNet
EORTC	European Organisation for Research and Treatment of Cancer
EQ-5D	EuroQol 5-Dimension Health Status Instrument
EQ-5D-5L	Five-level version of the EQ-5D
FACT-Leu	Functional Assessment of Cancer Therapy–Leukemia
HRQoL	Health-related quality of life
MDASI-CML	MD Anderson Symptom Inventory for CML
MID	Minimal important difference
MR	Molecular response
NCCN	National Comprehensive Cancer Network
OS	Overall survival
PROM	Patient-reported outcome measure
QLQ-C30	EORTC core quality-of-life questionnaire
QLQ-CML24	EORTC CML-specific quality-of-life module
QoL	Quality of life
SF-36	Short Form-36 Health Survey
TFR	Treatment-free remission
TKI	Tyrosine kinase inhibitor
